# FlsnRNA-seq: protoplasting-free full-length single-nucleus RNA profiling in plants

**DOI:** 10.1186/s13059-021-02288-0

**Published:** 2021-02-19

**Authors:** Yanping Long, Zhijian Liu, Jinbu Jia, Weipeng Mo, Liang Fang, Dongdong Lu, Bo Liu, Hong Zhang, Wei Chen, Jixian Zhai

**Affiliations:** 1 Department of Biology, School of Life Sciences, Southern University of Science and Technology, Shenzhen, 518055 China; 2 Institute of Plant and Food Science, Southern University of Science and Technology, Shenzhen, 518055 China; 3 Key Laboratory of Molecular Design for Plant Cell Factory of Guangdong Higher Education Institutes, Southern University of Science and Technology, Shenzhen, 518055 China; 4 Academy for Advanced Interdisciplinary Studies, Southern University of Science and Technology, Shenzhen, 518055 China

**Keywords:** Nanopore sequencing, Single-nucleus RNA-seq, Long-read

## Abstract

The broad application of single-cell RNA profiling in plants has been hindered by the prerequisite of protoplasting that requires digesting the cell walls from different types of plant tissues. Here, we present a protoplasting-free approach, flsnRNA-seq, for large-scale full-length RNA profiling at a single-nucleus level in plants using isolated nuclei. Combined with 10x Genomics and Nanopore long-read sequencing, we validate the robustness of this approach in *Arabidopsis* root cells and the developing endosperm. Sequencing results demonstrate that it allows for uncovering alternative splicing and polyadenylation-related RNA isoform information at the single-cell level, which facilitates characterizing cell identities.

**Supplementary Information:**

The online version contains supplementary material available at 10.1186/s13059-021-02288-0.

## Background

High-throughput single-cell transcriptome studies have thrived in animal and human research in recent years [1–5]. However, despite successful single-cell characterization at a relatively low scale in maize developing germ cells [6] and rice mesophyll cells [7] using capillary-based approaches [8], only a handful of large-scale single-cell RNA studies using high-throughput platforms such as 10x Genomics or Drop-seq [9] have been published in plants [10], most of which profiled protoplasts generated from the root of *Arabidopsis* [11–19]. A major reason for this narrow focus of tissue type is that plant cells are naturally confined by cell walls, and protoplasting is required to release individual cells—a procedure that is thoroughly tested for *Arabidopsis* roots [20–22] but remains to be difficult or impractical in many other tissues or species. Moreover, generating protoplasts from all cells uniformly is challenging given the complexity of plant tissues, and the enzymatic digestion and subsequent cleanup process during protoplast isolation may trigger the stress response and influence the transcriptome. Therefore, a protoplasting-free method is urgently needed to broaden the application of large-scale single-cell analysis in plants.

**Fig. 1:**
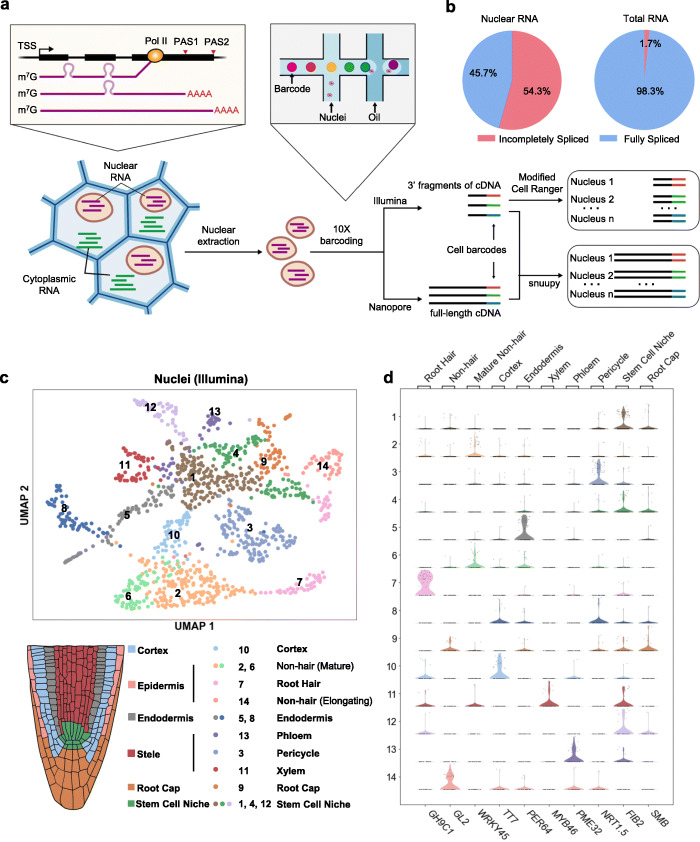
Protoplasting-free large-scale single-nucleus RNA-seq reveals the diverse cell types in *Arabidopsis* root. **a** Schematic diagram of protoplasting-free single-nucleus RNA-seq. **b** Incompletely spliced and fully spliced fractions of the Nanopore reads from our single-nucleus RNA library, compared with a previously published total RNA library (Parker et al., *eLife*, 2020). **c** UMAP visualization of the various cell types clustered using Illumina single-nucleus data (upper panel), and cartoon illustration of major cell types in *Arabidopsis* root tip (lower panel). **d** Violin plots showing the expression levels of previously reported cell type-specific marker genes in 14 clusters

We recently characterized full-length nascent RNAs in *Arabidopsis* and unexpectedly found a large number of polyadenylated mRNAs that are tightly associated with chromatin [23]. Since it is considerably easier and more widely applicable to perform nucleus isolation on various plant tissues than protoplasting, we set out to test if the polyadenylated RNAs in a single nucleus are sufficient to convey information on cell identity using the 10x Genomics high-throughput single-cell platform. Besides the standard Illumina short-read library which primarily captures abundance information, long-read sequencing has recently been incorporated into single-cell studies [24–26]. To access the large number of intron-containing RNAs in plant nuclei, we also constructed a Nanopore-based long-read library and developed a bioinformatic pipeline named “snuupy” (single nucleus utility in python) to characterize mRNA isoforms in each nucleus (Fig. 1a, Additional file 1: Fig. S1). Here, we applied the flsnRNA-seq to root and endosperm, respectively, and demonstrated that the long-read single-nucleus strategy would enable plant biologists to bypass protoplasting and study RNA isoforms derived from alternative splicing and alternative polyadenylation (APA) at the single-cell level and provides additional dimensions of transcriptome complexity that could potentially further improve clustering or characterization of different cell types.

## Results and discussion

First, we chose to use the *Arabidopsis* root to validate the effectiveness of our protoplasting-free single-nucleus RNA sequencing approach because of the well-studied cell types [27] and the rich resource of single-cell data [11–16] of this tissue. We directly isolated nuclei by sorting from homogenized root tips of 10-day-old *Arabidopsis* seedlings without protoplasting (Additional file 1: Fig. S2). The nuclei were fed to the 10x Genomics Chromium platform to obtain full-length cDNA templates labeled with nucleus-specific barcodes, which are subsequently divided into two equal parts and used for constructing Illumina short-read and Nanopore long-read libraries, respectively (Fig. 1a).

**Fig. 2:**
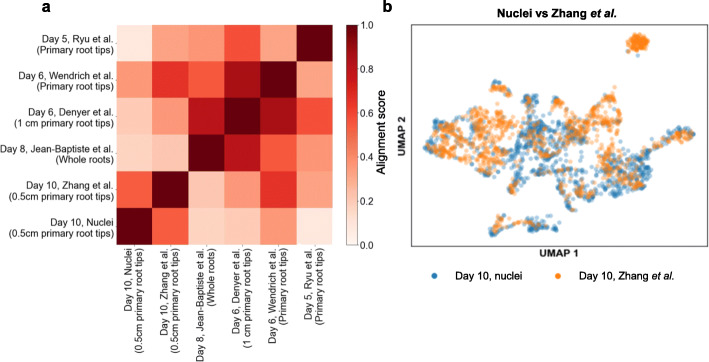
Dataset generated by flsnRNA-seq is consistent with protoplast-based scRNA-seq. **a** Heatmap represents alignment score between the single-nucleus data and single-cell datasets generated from 10x Genomics platform. Alignment score is calculated by Scanorama [30]. Higher alignment score indicates higher similarity between a pair of datasets. **b** Pairwise integration of two single cell/nucleus datasets. The batch effect is removed by Scanorama. The expression matrix is downsampled to the same dimension as the single-nucleus data

From the Illumina library, we obtained a total of 1186 single-nucleus transcriptomes covering 18,913 genes, with median genes/nucleus at 810 and median UMIs/nucleus at 1131. It is worth noting that the proportion of intron-containing mRNAs is extremely high in plant nucleus—54% compared to less than 2% in total RNAs [28] (Fig. 1b). After generating the cell-gene abundance matrix from Illumina data, we utilized an unbiased graph-based clustering method Louvain [29] and identified 14 distinct cell clusters (Fig. 1c). We then applied a set of cell type-specific marker genes provided in a recent massive single-cell study of *Arabidopsis* roots [17] to annotate each cluster (see the “Methods” section, Additional file 2: Table S1). We were able to assign cell types to all 14 clusters and identified 10 major root cell types previously reported (Fig. 1c, Additional file 1: Fig. S3), with the signature transcripts for each cell type enriched in the corresponding cluster (Fig. 1d, Additional file 1: Fig. S4). Consistent with previous reports [11–16], we also noticed that some cell types from our result are composed of multiple clusters, such as stem cell niche (clusters 1, 4, and 12), mature non-hair (clusters 2 and 6), and endodermis (clusters 5 and 8) (Fig. 1c), demonstrating additional heterogeneity (subcell types) within cell types. Moreover, we found the exact same subcell type marker genes of endodermis are enriched in each of its corresponding subcell types as shown in Zhang et al. [15] (Additional file 1: Fig. S5), demonstrating the robustness of our single-nucleus data. In addition, we used the Scanorama algorithm [30] to compare our dataset with several recently published root single-cell datasets from protoplasts [11, 12, 14–16]. The expression abundance matrix from our single-nucleus dataset closely resembles the protoplasting-based single-cell dataset generated from the same tissue (10-day seedling, 0.5 mm primary root tips) [15] (Fig. 2a, b). Taken together, we demonstrated that transcriptomes of the single nucleus are sufficient for cell type identification and can be used as a reliable alternative to protoplasts.

**Fig. 3:**
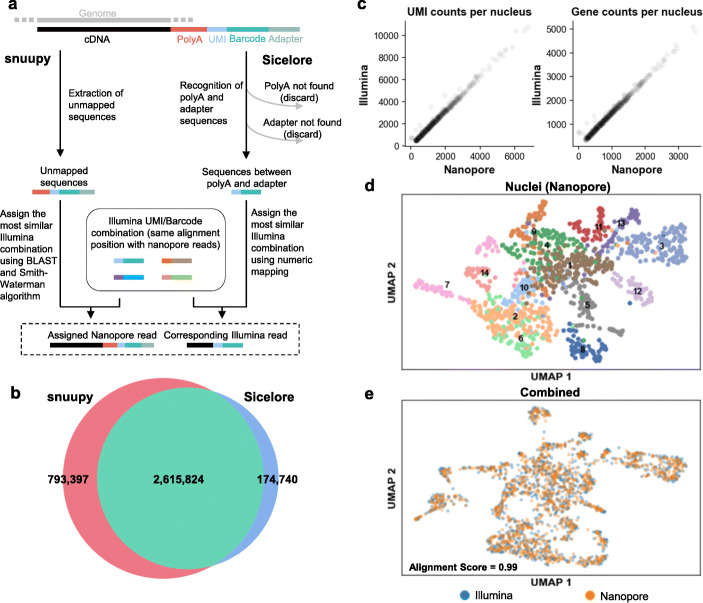
Snuupy assigns cell barcodes and UMIs for Nanopore reads according to the information from Illumina data. **a** Flowchart shows the difference between snuupy and Sicelore. **b** Overlap between snuupy and Sicelore allocated reads. **c** Numbers of UMIs (left) and genes (right) detected in each nucleus from the Illumina and Nanopore data. **d** UMAP visualization of the root cell types clustered using abundance information from the Nanopore single-nucleus data. The cell color is the same as in Fig. 1c. **e** UMAP visualization of the integration of two datasets. The batch effect is removed by Scanorama [30]. Alignment score is calculated by Scanorama and in the range from 0 to 1. Higher alignment score indicates a higher similarity between a pair of datasets

As to the Nanopore data analysis, a key challenge is that the relatively low sequencing accuracy of Nanopore (~ 95% per base) makes it difficult to correctly recognize the cell barcodes and UMI information on each Nanopore read. To solve this problem, Lebrigand et al. developed a method named Sicelore to use Illumina short reads generated from the same cDNA library as the guide to allocate Nanopore reads [24]. Sicelore searches for both polyA and adapter sequence and defines the region between these two as the potential barcode and UMI. However, this algorithm relies on the recognition of polyA tail sequence generated by the Nanopore basecalling software, which tends to severely underestimate the length of polyA tail [31]. We tried to further improve Sicelore by developing a polyA-independent algorithm (named snuupy), which searches for cell barcodes and UMIs in the unmapped region of Nanopore reads (see the “Methods” section and Additional file 1: Fig. S1, Fig. 3a). As the result, snuupy recovers 20% more reads from our Nanopore data compared to using Sicelore [24] (Fig. 3b). After snuupy processing, we obtained 1169 long-read single-nucleus transcriptomes from Nanopore data (compared to the 1186 from Illumina data). The median UMI counts per nucleus (729) and the median gene counts per nucleus (563) from Nanopore data are ~ 64% and ~ 70% of the Illumina count, respectively, and highly consistent in all nuclei (Fig. 3c). The clustering result using Nanopore abundance matrix closely resembles the one generated by Illumina data (Fig. 3d, e), suggesting that Nanopore data itself is sufficient for cell type classification, consistent with a recent large-scale single-cell analysis in human and mouse cells performed entirely with Nanopore data [24, 25].

**Fig. 4:**
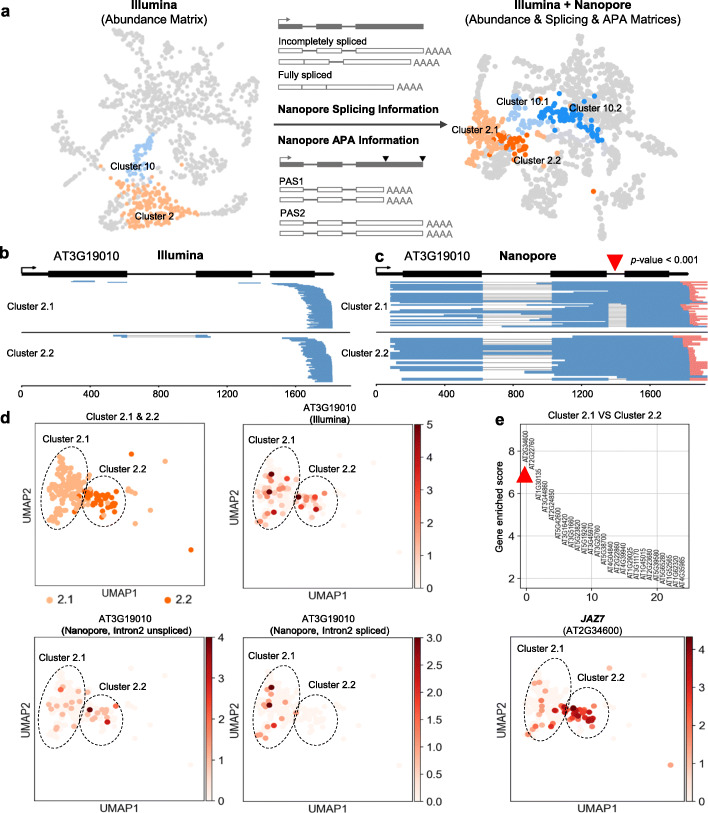
Nanopore long-read single-nucleus RNA-seq improves cell type identification. **a** Multilayer matrices combining Illumina abundance matrix with Nanopore splicing and APA information improve cell type identification. **b**, **c** Genome-browser plot of Illumina reads (**b**) and Nanopore reads (**c**) aligned to gene AT3G19010. The second intron of AT3G1910 shows different splicing patterns between cluster 2.1 and cluster 2.2. The red arrowhead indicates the second intron. Differences in splicing patterns between two clusters were tested using Fisher exact test, and the corresponding *p* value is lower than 0.001. The red bar at the 3′ end of Nanopore reads (blue) indicates the Poly(A) tail. **d** UMAP visualization shows the abundance distribution of AT3G19010 as well as the differential splicing of the second intron between cluster 2.1 and cluster 2.2. **e** The top 25 genes enriched in cluster 2.2 are ranked by enriched score compared to cluster 2.1 (upper panel) and UMAP visualization shows the abundance distribution of the most enriched gene *JAZ7* (lower panel). The enriched score is calculated using *rank_genes_groups function* of Scanpy. The red arrowhead indicates the most enriched gene in cluster 2.2

The single-nucleus long-read Nanopore library provides isoform-level information such as splicing and APA, compared to Illumina library which only captures abundance information of transcripts. Therefore, we generated two additional isoform matrices to track splicing and APA in single nucleus, respectively (Fig. 4a and Additional file 1: Fig. S6), and combined them with the Illumina abundance matrix for a multilayer clustering, to test if these extra layers of information could improve cell type classification. Indeed, we found that the original cluster 2 (mature non-hair) and cluster 10 (cortex) from Illumina data (Fig. 1c) can be further separated into two subcell type clusters after the multilayer clustering (Fig. 4a). As an example, from the Illumina data, transcripts of AT3G19010 are present in both subcell type 2.1 and 2.2 (Fig. 4b, c), while the Nanopore data revealed a large difference at the splicing level of this gene between the two sub-clusters, with the second intron largely unspliced in subcell type 2.2 (Fig. 4d). It is worth noting that, *JAZ7*, the top 1 enriched gene in cluster 2.2 (Fig. 4e), can regulate splicing during jasmonate response [32], implying a fascinating potential of cell type-specific regulation of splicing that could be investigated with flnsRNA-seq.

**Fig. 5:**
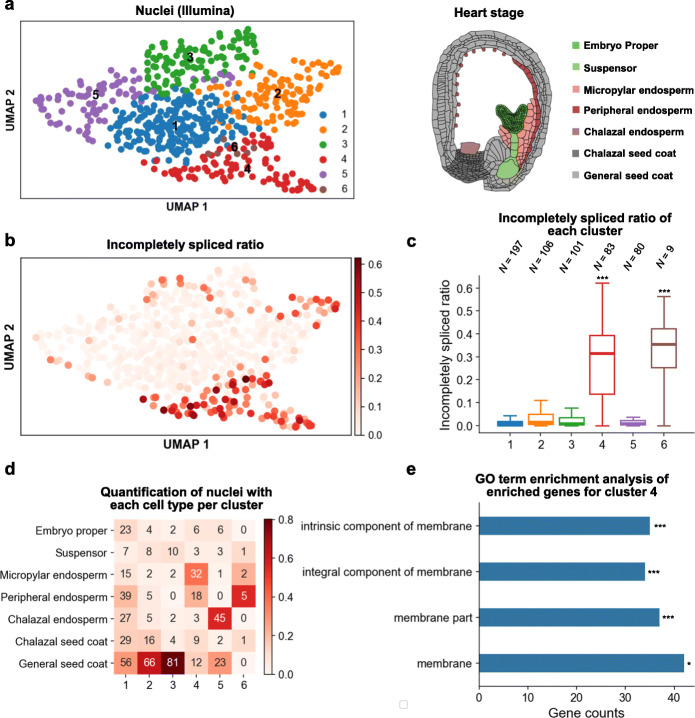
flsnRNA-seq captures the variation in intron retention levels of different clusters. **a** UMAP visualization of clustering result using Illumina single-nucleus data (left panel), and cartoon illustration of major cell types in *Arabidopsis* endosperm at heart stage (right panel). **b** UMAP visualization of incompletely spliced ratio calculated by Nanopore full-length reads. **c** Barplot visualization of the incompletely spliced ratio of each cluster. Differences in incompletely spliced ratios between each cluster to all other clusters were tested using a one-sided Kolmogorov-Smirnov test. “***” denotes that the *p* value is lower than 0.001. **d** Quantification of nuclei with each cell type per cluster. The number represents nucleus counts and the color represents the proportion of cell types in each cluster. **e** GO term enrichment analysis of all 93 enriched genes for cluster 4. Only cellular component terms are plotted. “*” and “***” denote that the adjusted *p* value is lower than 0.05 and 0.001, respectively

After establishing flsnRNA-seq using well-documented root tissue, we applied this method to investigate other tissues that have not been previously characterized at the single-cell level due to difficulties in obtaining corresponding protoplasts. In flowering plants, seed development is initiated by double fertilization, during which egg cell and central cell fuse with sperm cells respectively to form embryo and endosperm [33]. The endosperm is embedded in the seed coat and responsible for providing nutrients from maternal parent to developing embryo [34, 35]. In *Arabidopsis* endosperm, the primary nucleus formed after fertilization undergoes several rounds of rapid nuclear divisions without cytokinesis, resulting in a multinucleate cell termed syncytium, which later cellularized and differentiated into three endosperm domains: the micropylar, central peripheral, and chalazal [36, 37] (Fig. 5a). Cellularization of the syncytium is critical for embryo viability [34], and this process is initiated when the embryo reaches the heart stage, starting from the micropylar domain and gradually proceeds to the central periphery in a wavelike pattern and eventually reach the chalazal zone [38]. Transcriptomes from various developing stages of bulked endosperm have been well-documented using microarray or RNA-seq [39–45]; however, endosperm has yet to be characterized at the single-cell level due to technical challenges in generating protoplasts from the endosperm.

Here, we applied flsnRNA-seq to the multinucleate endosperm isolated from the heart-stage ovules of *Arabidopsis* and generated both the Illumina and Nanopore 10x libraries. We obtained 576 nuclei from Illumina data, with the median genes/nucleus at 645 and the median UMIs/nucleus at 853. All 576 nuclei were captured by the Nanopore library, with the median genes/nucleus at 300 and the median UMIs/nucleus at 362 (Additional file 1: Fig. S7). Based on the Illumina abundance matrix, we identified six clusters using the Louvain method (Fig. 5a). Next, we used Nanopore full-length transcript data to analyze retained introns in each nucleus and found that the nuclei from cluster 4, a major cluster that accounts for 14% of the total nuclei, exhibits a distinct high ratio of incompletely spliced transcripts (Fig. 5b, c). Several previous studies have established that increased accumulation of intronic reads is an indicator of transcription activation [46, 47], and the ratio of unspliced precursor and spliced mature transcripts have been widely used to estimate RNA velocity [48, 49]. The high ratio of incompletely spliced transcripts in this particular cluster of endosperm nuclei may reflect delayed pre-mRNA decay, disrupted intron turnover rate, or a global activation of transcription. Next, we assigned the cell type of each nucleus using previously reported cell type-enriched genes at the heart stage [50, 51] and found that the majority of nuclei in cluster 4 are annotated as micropylar endosperm (Fig. 5d). In addition, gene ontology (GO) analysis of the genes upregulated in cluster 4 found all enriched cellular component terms are membrane related (Fig. 5e, Additional file 2: Table S3), suggesting these nuclei are poised to forming the cellular membrane and entering the cellularization stage, consistent with the previous observation that cellularization of the *Arabidopsis* endosperm is first initiated at the micropylar endosperm [38, 52]. Hence, our method identified a unique cluster of endosperm nuclei with a high proportion of incompletely spliced transcripts, and further investigation could determine whether this is due to the increase in transcription or delay in splicing.

## Conclusion

Transcriptome profiling using single nuclei of neurons or frozen tissues has been well-established in animals, and the result is comparable to using the RNA from single cells [53–56]. In plants, the transcriptome from isolated nuclei and from total RNA of the endosperm has also been shown to be well-correlated [41]. Here, as a proof-of-concept demonstration in plant, our results showed that protoplasting-free large-scale single-nucleus sequencing is sufficient for cell type classification and marker gene identification in *Arabidopsis*. The cost per cell for single-cell sequencing using isolated nuclei on the 10x Genomics platform is still relatively higher compared to using protoplast due to lower capturing efficiency of 10x Genomics on nuclei versus cell, possibly due to the much smaller size of nucleus; thus, this step still could benefit from further optimization. As we are preparing this manuscript, several groups have also recently adopted the nucleus-based protoplasting-free strategy independently to investigate a wide range of plant tissues, including root, leaf, seedling, shoot apex, endosperm, and inflorescence [57–61]. Eliminating protoplasting as a prerequisite would enable large-scale single-cell profiling on a wide range of tissues and plant species; nevertheless, isolating nuclei from some cell types remains challenging and requires further developments of the nucleus isolation protocol [57]. Our method uniquely combined the Nanopore-based full-length RNA sequencing method with single-nucleus sequencing to capture isoform diversity at the single-nucleus level, which can facilitate cell type classification and characterization by providing extra layers of information in addition to abundance.

## Methods

### Nucleus isolation from root tip of *Arabidopsis*

The wild-type *Arabidopsis* seedlings (Col-0) were grown on 1/2 MS plates at 22 °C (16 h light/8 h dark) for 10 days before harvest. The root tip region (5 mm) of seedlings were cut and transferred immediately into a 1.5-ml RNase-free Eppendorf tube kept in liquid nitrogen and were ground into fine powder by a 1000-μl pipette tip in the tube. The powder was then dissolved in 300 μl ice-cold Extraction Buffer (EB)—0.4 M sucrose, 10 mM Tris-HCl pH 8.0, 10 mM MgCl_2_, 0.2% (w/v) Triton X-100, 1 mM dithiothreitol (DTT), 1× protease inhibitor (Roche), 0.4 U/μl RNase inhibitor (RNaseOUT, Thermo Fisher Scientific). Nonionic surfactant Triton X-100 is used to release nuclei and avoid aggregation during FACS [62]. After gentle vertexing and inversion, the homogenate was filtered through a 20-μm cell strainer into a new tube. Another 400 μl EB was added to the strainer to wash the remaining nuclei. After centrifugation at 4 °C, 2000*g* for 5 min, the supernatant was removed carefully to avoid RNA contaminants from the cytoplasmic fraction. The pellet was washed twice at 4 °C, 2000*g*, 5 min with 1 ml EB, and then resuspended in 500 μl EB. For sorting, the nuclei were stained with 4,6-diamidino-2-phenylindole (DAPI) and loaded into a flow cytometer with a 70-μm nozzle. One-milliliter EB was used as the collection buffer. A total of 40,000 nuclei were sorted based on the DAPI signal and the nuclear size. To avoid aggregation, the sorted nuclei were pelleted at 4 °C, 2000*g*, 5 min, and then resuspended in 100 μl PBST buffer (1× PBS with a low concentration of 0.025% Triton X-100). After checking the quality of nuclei and counting under a microscope using the DAPI channel, 5000 nuclei were transferred into a new tube with 500 μl PBST buffer and centrifuged at 4 °C, 2000*g*, 5 min. Then, the pellet was resuspended in 20 μl PBST buffer and diluted to about 1000 nuclei/μl.

### Endosperm nucleus isolation

Endosperm was isolated as previously reported [63, 64]. Briefly, 5 DAP ovules at the heart stage of embryogenesis were placed on a slide under a dissecting microscope. The endosperm nuclei were released to the slide from the seed by gentle pressure after piercing by a needle. The nuclei were resuspended in isolation buffer (0.3 M sorbitol, 5 mM MES (pH 5.7), 0.4 U/μl RNaseOUT) and transferred to a low-bound 1.5-ml Eppendorf tube by a 10-μl pipette. Then, the collected nuclei were filtered with a 20-μm cell strainer to a new tube and then washed twice with washing buffer (EB) at 4 °C, 2000*g*, 5 min to remove free RNA and contaminants. Then, the nuclei were resuspended in 100 μl PBST buffer and diluted to 1000 nuclei/μl.

### Single-nucleus RNA-seq library construction for Illumina and Nanopore sequencing

Libraries were constructed according to the standard 10x Genomics protocol (Single Cell 3′ Reagent Kits v2 User Guide) with modifications to accommodate Nanopore long-read sequencing. For root tip, nucleus suspension from the previous step (~ 5000 nuclei) were loaded onto the 10x Genomics ChIP, and libraries were made using a 10x Chromium Single Cell 3′ Solution V2 kit. For endosperm, ~ 10,000 nuclei were loaded and subjected to library construction. To obtain full-length cDNA, we extend the elongation time during cDNA amplification from the standard 1 min to 2 min. Half of the cDNA template was used to construct Illumina library according to the manufacturer’s instruction and sequenced with Illumina NavoSeq (Read1:28 bases + Read2:150 bases); the other half of the template was used to make Nanopore library using the Oxford Nanopore LSK-109 kit and sequenced on a MinION flow cell (R9.4.1).

### Illumina single-nucleus data analysis

Raw reads of root were mapped to the TAIR10 reference genome by Cell Ranger (v3.1.0) using the default parameters. Cell Ranger (v3.1.0) only counts reads without introns; to accommodate the high proportion of intron-containing reads in our single-nucleus libraries, we removed the intron regions of each read and re-aligned reads to the reference genome by Cell Ranger to identify the nucleus barcode, UMI, and corresponding gene of each read (Additional file 1: Fig. S1). For quality control purpose, genes expressed in less than three nuclei were discarded, and cells with gene counts more than 2300 or fewer than 350 were removed. The Illumina abundance matrix was subsequently analyzed using Scanpy package (v1.6.0) [65] with recommended parameters for normalization, log-transformation, and scaling. Then, principal component analysis and Louvain algorithm were used on this abundance matrix for clustering. Next, we used the marker genes for different cell types identified in a massive single-cell root data [17] (Additional file 2: Table S1) to annotate the cell type of each cluster. We first calculate the cell score of each cell type for all cells based on the enrichment degree of a given marker gene set in a given cell, as previously described method [66]. If the highest score exceeds zero, the cell is assigned to the corresponding cell type; otherwise, it is assigned as unknown (Additional file 1: Fig. S3a). Then, each cluster was annotated as the cell type with the highest proportion (Additional file 1: Fig. S3b), and we used developmental stage-specific genes identified in the massive single-cell root data [17] (Additional file 1: Table S1) to further annotate the clusters resenting non-hair cells as either mature non-hair or elongating non-hair cells (Additional file 1: Fig. S3c).

Five previously published single-cell RNA-seq data of protoplasted *Arabidopsis* roots using 10x Genomics platform were collected from public databases [11, 12, 14–16]. We use Scanorama [30] to remove batch effects and calculate the alignment score between different datasets.

For endosperm data, raw Illumina reads were processed by Cell Ranger (v5.0.0) using the “--include-introns” parameter, and only nuclei with gene counts between 400 and 3000 were used for the subsequent analysis. After clustering, we used previously reported heart-stage tissue-enriched genes identified from the microarray data of laser capture microdissection samples [50, 51] to assign cell types (Additional file 2: Table S2). We use the “rank_genes_groups” function of scanpy to perform the Wilcoxon test and used “filter_rank_genes_groups” function with “max_out_group_fraction = 0.25” and “min_ fold_change = 1.50” to identify cluster-enriched genes. And agriGO v2 [67] was used for GO enrichment analysis.

### Nanopore single-nucleus data processing and isoform analysis

Raw Nanopore data were basecalled using Guppy (v3.6.0) with the parameters “--c dna_r9.4.1_450bps_hac.cfg --fast5_out.” The basecalled reads were mapped to the TAIR10 genome by minimap2 (v2.17) with the parameters “-ax splice --secondary=no -uf --MD --sam-hit-only,” and the multi-mapped reads as well as potential chimeric reads (either the 5′ or 3′ unmapped region is great than 150 nt) were filtered out. The nucleus barcodes and UMI sequences in Nanopore reads were extracted from the unmapped sequences of each read via aligning against all barcode/UMI combinations identified from the Illumina library made from the same full-length cDNA templates, a strategy inspired by the algorithm Sicelore [24]. To reduce search space, we divided the genome into non-overlapped 500-bp bins, and only matched the Illumina barcode/UMI combinations from the bins overlapping or adjacent to the mapping genome region of specific Nanopore read (Fig. 3a). To speed up the alignment process, we first used the heuristic algorithm Blastn (v2.10.0) to find potential seed regions with parameters “-word_size 7 -gapopen 0 -gapextend 2 -penalty -1 -reward 1” and then re-aligned the seed regions by the more accurate Smith-Waterman local alignment algorithm. Our pipeline assigns the closest barcode-UMI match (i.e., with minimal mismatch/gap) to each Nanopore read, allowing up to three base errors (mismatch/gap) for either barcode or UMI, and remove reads with multiple best matching barcode-UMIs. After the barcode and UMI assignment, the Nanopore reads with the same UMI were used to generate an error-corrected consensus sequence of the original RNA molecule by poaV2 [68] and racon [69]. PAS isoform annotation and the intron splicing status of Nanopore read were determined based on previously described [23, 70]. In brief, we clustered adjacent polyA sites into one polyA site cluster with a distance threshold 24 nt and then count the reads in each polyA site cluster to obtain the APA matrix. And we defined the reads containing at least one intron with a mapping ratio of more than 50% as unspliced read, and the others were defined as spliced read. The resulted APA and splicing matrices for all nuclei were merged with Illumina abundance matrix and analyzed by Scanpy.

The same Cell Ranger result is used as the input file for Sicelore. Except that the maximum edit distance during barcode and UMI assignment is forcibly set to 3, the remaining parameters are the same as the official example (https://github.com/ucagenomix/sicelore/blob/master/quickrun.sh).

## Supplementary Information

**Additional file 1: Fig. S1.** Schematic diagram of snuupy bioinformatic pipeline. **Fig. S2.** The sorted nuclei were observed under a microscopy with DAPI staining. **Fig. S3.** Identification of clusters by a marker-gene-based method. **Fig. S4.** UMAP visualization of the representative cell-type marker genes for each of the 14 cell clusters. **Fig. S5.** UMAP visualization showing the abundances of representative marker genes in two subcell types of endodermis. **Fig. S6.** Scheme for deriving the splicing and APA matrices from Nanopore data. **Fig. S7.** The gene expression matrices of endosperm generated from the two different libraries were similar to each other. (PPTX 4884 kb)

**Additional file 2: Table S1.** Cell type-specific genes identified by Shahan et al. are used for cell type annotation. **Table S2.** Cell type-enriched genes at heart stage identified by Schon and Nodine are used for cell type annotation. **Table S3.** All enriched genes for cluster 4.

**Additional file 3.** Review history.

## Data Availability

Flsn-seq data generated in this study are deposited in NCBI with the accession numbers PRJNA664874 (Root) [71] and PRJNA685588 (Endosperm) [72]. The preprocessed datasets analyzed in the study and the source code can be downloaded from Zenodo (10.5281/zenodo.4467583) [73] or GitHub repository (https://github.com/ZhaiLab-SUSTech/snuupy/tree/master) [74].

## References

[1] Chen X, Teichmann SA, Meyer KB (2018). From tissues to cell types and back: single-cell gene expression analysis of tissue architecture. Annu Rev Biomed Data Sci.

[2] Lein E, Borm LE, Linnarsson S (2017). The promise of spatial transcriptomics for neuroscience in the era of molecular cell typing. Science.

[3] Kelsey G, Stegle O, Reik W (2017). Single-cell epigenomics: recording the past and predicting the future. Science.

[4] Stubbington MJT, Rozenblatt-Rosen O, Regev A, Teichmann SA (2017). Single-cell transcriptomics to explore the immune system in health and disease. Science.

[5] Svensson V, Vento-Tormo R, Teichmann SA (2018). Exponential scaling of single-cell RNA-seq in the past decade. Nat Protoc.

[6] Nelms B, Walbot V (2019). Defining the developmental program leading to meiosis in maize. Science.

[7] Han Y, Chu X, Yu H, Ma Y-K, Wang X-J, Qian W, Jiao Y (2017). Single-cell transcriptome analysis reveals widespread monoallelic gene expression in individual rice mesophyll cells. Sci Bull.

[8] Luo C, Fernie AR, Yan J (2020). Single-cell genomics and epigenomics: technologies and applications in plants. Trends Plant Sci.

[9] Macosko Evan Z, Basu A, Satija R, Nemesh J, Shekhar K, Goldman M, Tirosh I, Bialas Allison R, Kamitaki N, Martersteck Emily M (2015). Highly parallel genome-wide expression profiling of individual cells using nanoliter droplets. Cell.

[10] Rich-Griffin C, Stechemesser A, Finch J, Lucas E, Ott S, Schäfer P (2020). Single-cell transcriptomics: a high-resolution avenue for plant functional genomics. Trends Plant Sci.

[11] Denyer T, Ma X, Klesen S, Scacchi E, Nieselt K, Timmermans MCP (2019). Spatiotemporal developmental trajectories in the *Arabidopsis* root revealed using high-throughput single-cell RNA sequencing. Dev. Cell.

[12] Jean-Baptiste K, McFaline-Figueroa JL, Alexandre CM, Dorrity MW, Saunders L, Bubb KL, Trapnell C, Fields S, Queitsch C, Cuperus JT (2019). Dynamics of gene expression in single root cells of *Arabidopsis thaliana*. Plant Cell.

[13] Shulse CN, Cole BJ, Ciobanu D, Lin J, Yoshinaga Y, Gouran M, Turco GM, Zhu Y, O’Malley RC, Brady SM, Dickel DE (2019). High-throughput single-cell transcriptome profiling of plant cell types. Cell Rep..

[14] Ryu KH, Huang L, Kang HM, Schiefelbein J (2019). Single-cell RNA sequencing resolves molecular relationships among individual plant cells. Plant Physiol.

[15] Zhang T-Q, Xu Z-G, Shang G-D, Wang J-W (2019). A single-cell RNA sequencing profiles the developmental landscape of *Arabidopsis* root. Mol Plant.

[16] Wendrich JR, Yang B, Vandamme N, Verstaen K, Smet W, Van de Velde C, Minne M, Wybouw B, Mor E, Arents HE (2020). Vascular transcription factors guide plant epidermal responses to limiting phosphate conditions. Science.

[17] Shahan R, Hsu C-W, Nolan TM, Cole BJ, Taylor IW, Vlot AHC, Benfey PN, Ohler U: A single cell *Arabidopsis* root atlas reveals developmental trajectories in wild type and cell identity mutants. bioRxiv 2020:2020.2006.2029.178863. 10.1016/j.devcel.2022.01.008 PMC9014886 35134336

[18] Liu Q, Liang Z, Feng D, Jiang S, Wang Y, Du Z, Li R, Hu G, Zhang P, Ma Y, et al. Transcriptional landscape of rice roots at the single cell resolution. Mol Plant. 2020; 10.1016/j.molp.2020.12.014. 10.1016/j.molp.2020.12.014 33352304

[19] Satterlee JW, Strable J, Scanlon MJ. Plant stem-cell organization and differentiation at single-cell resolution. Proc Natl Acad Sci U S A. 2020;117:33689-99. 10.1073/pnas.2018788117 PMC7776995 33318187

[20] Birnbaum K, Shasha DE, Wang JY, Jung JW, Lambert GM, Galbraith DW, Benfey PN (2003). A gene expression map of the *Arabidopsis* root. Science.

[21] Brady SM, Orlando DA, Lee J-Y, Wang JY, Koch J, Dinneny JR, Mace D, Ohler U, Benfey PN (2007). A high-resolution root spatiotemporal map reveals dominant expression patterns. Science.

[22] Li S, Yamada M, Han X, Ohler U, Benfey Philip N (2016). High-resolution expression map of the *Arabidopsis* root reveals alternative splicing and lincRNA regulation. Dev Cell.

[23] Jia J, Long Y, Zhang H, Li Z, Liu Z, Zhao Y, Lu D, Jin X, Deng X, Xia R (2020). Post-transcriptional splicing of nascent RNA contributes to widespread intron retention in plants. Nat Plants.

[24] Lebrigand K, Magnone V, Barbry P, Waldmann R (2020). High throughput error corrected Nanopore single cell transcriptome sequencing. Nat Commun.

[25] Volden R, Vollmers C: Highly multiplexed single-cell full-length cDNA sequencing of human immune cells with 10x Genomics and R2C2. bioRxiv 2020:2020.2001.2010.902361.

[26] Gupta I, Collier PG, Haase B, Mahfouz A, Joglekar A, Floyd T, Koopmans F, Barres B, Smit AB, Sloan SA (2018). Single-cell isoform RNA sequencing characterizes isoforms in thousands of cerebellar cells. Nat Biotechnol.

[27] Drapek C, Sparks EE, Benfey PN (2017). Uncovering gene regulatory networks controlling plant cell differentiation. Trends Genet.

[28] Parker MT, Knop K, Sherwood AV, Schurch NJ, Mackinnon K, Gould PD, Hall AJW, Barton GJ, Simpson GG (2020). Nanopore direct RNA sequencing maps the complexity of *Arabidopsis* mRNA processing and m6A modification. eLife.

[29] Blondel VD, Guillaume J-L, Lambiotte R, Lefebvre E (2008). Fast unfolding of communities in large networks. J Stat Mech Theory Exp.

[30] Hie B, Bryson B, Berger B (2019). Efficient integration of heterogeneous single-cell transcriptomes using Scanorama. Nat Biotechnol.

[31] Krause M, Niazi AM, Labun K, Torres Cleuren YN, Müller FS, Valen E (2019). tailfindr: alignment-free poly(A) length measurement for Oxford Nanopore RNA and DNA sequencing. RNA.

[32] Feng G, Yoo M-J, Davenport R, Boatwright JL, Koh J, Chen S, Barbazuk WB (2020). Jasmonate induced alternative splicing responses in *Arabidopsis*. Plant Direct.

[33] Bleckmann A, Alter S, Dresselhaus T. The beginning of a seed: regulatory mechanisms of double fertilization. Front Plant Sci. 2014;5:452. 10.3389/fpls.2014.00452 PMC4160995 25309552

[34] Lafon-Placette C, Köhler C (2014). Embryo and endosperm, partners in seed development. Curr Opin Plant Biol.

[35] Gehring M, Choi Y, Fischer RL (2004). Imprinting and seed development. Plant Cell.

[36] Sorensen MB, Mayer U, Lukowitz W, Robert H, Chambrier P, Jurgens G, Somerville C, Lepiniec L, Berger F (2002). Cellularisation in the endosperm of *Arabidopsis thaliana* is coupled to mitosis and shares multiple components with cytokinesis. Development.

[37] Brown RC, Lemmon BE, Nguyen H (2003). Events during the first four rounds of mitosis establish three developmental domains in the syncytial endosperm of *Arabidopsis thaliana*. Protoplasma.

[38] Brown RC, Lemmon BE, Nguyen H, Olsen O-A (1999). Development of endosperm in *Arabidopsis thaliana*. Sex Plant Reprod.

[39] Gehring M, Missirian V, Henikoff S (2011). Genomic analysis of parent-of-origin allelic expression in *Arabidopsis thaliana* seeds. PLoS One.

[40] Hsieh T-F, Shin J, Uzawa R, Silva P, Cohen S, Bauer MJ, Hashimoto M, Kirkbride RC, Harada JJ, Zilberman D, Fischer RL (2011). Regulation of imprinted gene expression in *Arabidopsis* endosperm. Proc Natl Acad Sci U S A.

[41] Del Toro-De León G, Köhler C (2019). Endosperm-specific transcriptome analysis by applying the INTACT system. Plant Reprod..

[42] Day RC, Herridge RP, Ambrose BA, Macknight RC (2008). Transcriptome analysis of proliferating *Arabidopsis* endosperm reveals biological implications for the control of syncytial division, cytokinin signaling, and gene expression regulation. Plant Physiol.

[43] Pignatta D, Erdmann RM, Scheer E, Picard CL, Bell GW, Gehring M (2014). Natural epigenetic polymorphisms lead to intraspecific variation in *Arabidopsis* gene imprinting. eLife.

[44] Moreno-Romero J, Santos-González J, Hennig L, Köhler C (2017). Applying the INTACT method to purify endosperm nuclei and to generate parental-specific epigenome profiles. Nat Protoc.

[45] Moreno-Romero J, Del Toro-De León G, Yadav VK, Santos-González J, Köhler C (2019). Epigenetic signatures associated with imprinted paternally expressed genes in the *Arabidopsis* endosperm. Genome Biol.

[46] Zeisel A, Köstler WJ, Molotski N, Tsai JM, Krauthgamer R, Jacob-Hirsch J, Rechavi G, Soen Y, Jung S, Yarden Y, Domany E (2011). Coupled pre-mRNA and mRNA dynamics unveil operational strategies underlying transcriptional responses to stimuli. Mol Syst Biol.

[47] Gaidatzis D, Burger L, Florescu M, Stadler MB (2015). Analysis of intronic and exonic reads in RNA-seq data characterizes transcriptional and post-transcriptional regulation. Nat Biotechnol.

[48] La Manno G, Soldatov R, Zeisel A, Braun E, Hochgerner H, Petukhov V, Lidschreiber K, Kastriti ME, Lönnerberg P, Furlan A (2018). RNA velocity of single cells. Nature.

[49] Bergen V, Lange M, Peidli S, Wolf FA, Theis FJ (2020). Generalizing RNA velocity to transient cell states through dynamical modeling. Nat Biotechnol.

[50] Schon MA, Nodine MD (2017). Widespread contamination of *Arabidopsis* embryo and endosperm transcriptome data sets. Plant Cell.

[51] Belmonte MF, Kirkbride RC, Stone SL, Pelletier JM, Bui AQ, Yeung EC, Hashimoto M, Fei J, Harada CM, Munoz MD (2013). Comprehensive developmental profiles of gene activity in regions and subregions of the *Arabidopsis* seed. Proc Natl Acad Sci U S A.

[52] Orozco-Arroyo G, Paolo D, Ezquer I, Colombo L (2015). Networks controlling seed size in *Arabidopsis*. Plant Reprod.

[53] Habib N, Li Y, Heidenreich M, Swiech L, Avraham-Davidi I, Trombetta JJ, Hession C, Zhang F, Regev A (2016). Div-Seq: single-nucleus RNA-Seq reveals dynamics of rare adult newborn neurons. Science.

[54] Habib N, Avraham-Davidi I, Basu A, Burks T, Shekhar K, Hofree M, Choudhury SR, Aguet F, Gelfand E, Ardlie K (2017). Massively parallel single-nucleus RNA-seq with DroNc-seq. Nat Methods.

[55] Ding J, Adiconis X, Simmons SK, Kowalczyk MS, Hession CC, Marjanovic ND, Hughes TK, Wadsworth MH, Burks T, Nguyen LT (2020). Systematic comparison of single-cell and single-nucleus RNA-sequencing methods. Nat Biotechnol.

[56] Krienen FM, Goldman M, Zhang Q, C. H. del Rosario R, Florio M, Machold R, Saunders A, Levandowski K, Zaniewski H, Schuman B, et al: Innovations present in the primate interneuron repertoire. Nature 2020, 586:262–269. 10.1038/s41586-020-2781-z PMC7957574 32999462

[57] Thibivilliers S, Anderson D, Libault M (2020). Isolation of plant root nuclei for single cell RNA sequencing. Curr Protoc Plant Biol.

[58] Picard CL, Povilus RA, Williams BP, Gehring M: Single nucleus analysis of *Arabidopsis* seeds reveals new cell types and imprinting dynamics. bioRxiv 2020:2020.2008.2025.267476.

[59] Tian C, Du Q, Xu M, Du F, Jiao Y: Single-nucleus RNA-seq resolves spatiotemporal developmental trajectories in the tomato shoot apex. bioRxiv 2020:2020.2009.2020.305029.

[60] Farmer A, Thibivilliers S, Ryu KH, Schiefelbein J, Libault M. Single-nucleus RNA and ATAC sequencing reveals the impact of chromatin accessibility on gene expression in Arabidopsis roots at the single-cell level. Mol Plant. 2021; 10.1016/j.molp.2021.01.001. 10.1016/j.molp.2021.01.001 33422696

[61] Sunaga-Franze DY, Muino JM, Braeuning C, Xu X, Zong M, Smaczniak C, Yan W, Fischer C, Vidal R, Kliem M, et al: Single-nuclei RNA-sequencing of plants. bioRxiv 2020:2020.2011.2014.382812. 10.1111/tpj.15458 34390289

[62] Krishnaswami SR, Grindberg RV, Novotny M, Venepally P, Lacar B, Bhutani K, Linker SB, Pham S, Erwin JA, Miller JA (2016). Using single nuclei for RNA-seq to capture the transcriptome of postmortem neurons. Nat Protoc.

[63] Gehring M, Huh JH, Hsieh TF, Penterman J, Choi Y, Harada JJ, Goldberg RB, Fischer RL (2006). DEMETER DNA glycosylase establishes *MEDEA* polycomb gene self-imprinting by allele-specific demethylation. Cell.

[64] Ibarra CA, Feng X, Schoft VK, Hsieh TF, Uzawa R, Rodrigues JA, Zemach A, Chumak N, Machlicova A, Nishimura T (2012). Active DNA demethylation in plant companion cells reinforces transposon methylation in gametes. Science.

[65] Wolf FA, Angerer P, Theis FJ (2018). SCANPY: large-scale single-cell gene expression data analysis. Genome Biol.

[66] Tirosh I, Izar B, Prakadan SM, Wadsworth MH, Treacy D, Trombetta JJ, Rotem A, Rodman C, Lian C, Murphy G (2016). Dissecting the multicellular ecosystem of metastatic melanoma by single-cell RNA-seq. Science.

[67] Tian T, Liu Y, Yan H, You Q, Yi X, Du Z, Xu W, Su Z (2017). agriGO v2.0: a GO analysis toolkit for the agricultural community, 2017 update. Nucleic Acids Res.

[68] Lee C, Grasso C, Sharlow MF (2002). Multiple sequence alignment using partial order graphs. Bioinformatics.

[69] Vaser R, Sović I, Nagarajan N, Šikić M (2017). Fast and accurate de novo genome assembly from long uncorrected reads. Genome Res.

[70] Wu X, Liu M, Downie B, Liang C, Ji G, Li QQ, Hunt AG (2011). Genome-wide landscape of polyadenylation in *Arabidopsis* provides evidence for extensive alternative polyadenylation. Proc Natl Acad Sci U S A.

[71] Long Y, Liu Z, Jia J, Mo W, Fang L, Lu D, Liu B, Zhang H, Chen W, Zhai J. A protoplasting-free approach for high-throughput full-length single-cell RNA profiling in plants. Datasets. Gene Expression Omnibus. https://www.ncbi.nlm.nih.gov/bioproject/PRJNA664874 (2020). Accessed 21 Sep 2020.

[72] Long Y, Liu Z, Jia J, Mo W, Fang L, Lu D, Liu B, Zhang H, Chen W, Zhai J. Single-nucleus full-length RNA profiling of endosperm. Datasets. Gene Expression Omnibus. https://www.ncbi.nlm.nih.gov/bioproject/PRJNA685588 (2021). Accessed 16 Dec 2020.

[73] Long Y, Liu Z, Jia J, Mo W, Fang L, Lu D, Liu B, Zhang H, Chen W, Zhai J. Single-nucleus nanopore reads processing pipeline. zenode. 10.5281/zenodo.4467583 (2021). Accessed 26 Jan 2021.

[74] Long Y, Liu Z, Jia J, Mo W, Fang L, Lu D, Liu B, Zhang H, Chen W, Zhai J. Single-nucleus nanopore reads processing pipeline. Github. https://github.com/ZhaiLab-SUSTech/snuupy/tree/master (2021). Accessed 26 Jan 2021.

