# Supplementary material for: FlsnRNA-seq: protoplasting-free full-length single-nucleus RNA profiling in plants
Source: Genome Biol. 2021 Feb 19;22:66. doi: 10.1186/s13059-021-02288-0 (PMC7893963; doi:10.1186/s13059-021-02288-0)
Supplement: Supplementary file 1 — Additional file 1: Fig. S1. Schematic diagram of snuupy bioinformatic pipeline. Fig. S2. The sorted nuclei were observed under a microscopy with DAPI staining. Fig. S3. Identification of clusters by a marker-gene-based method. Fig. S4. UMAP visualization of the representative cell-type marker genes for each of the 14 cell clusters. Fig. S5. UMAP visualization showing the abundances of representative marker genes in two subcell types of endodermis. Fig. S6. Scheme for deriving the splicing and APA matrices from Nanopore data. Fig. S7. The gene expression matrices of endosperm generated from the two different libraries were similar to each other. (PPTX 4884 kb) [file 13059_2021_2288_MOESM1_ESM.pptx]

## Slide 1
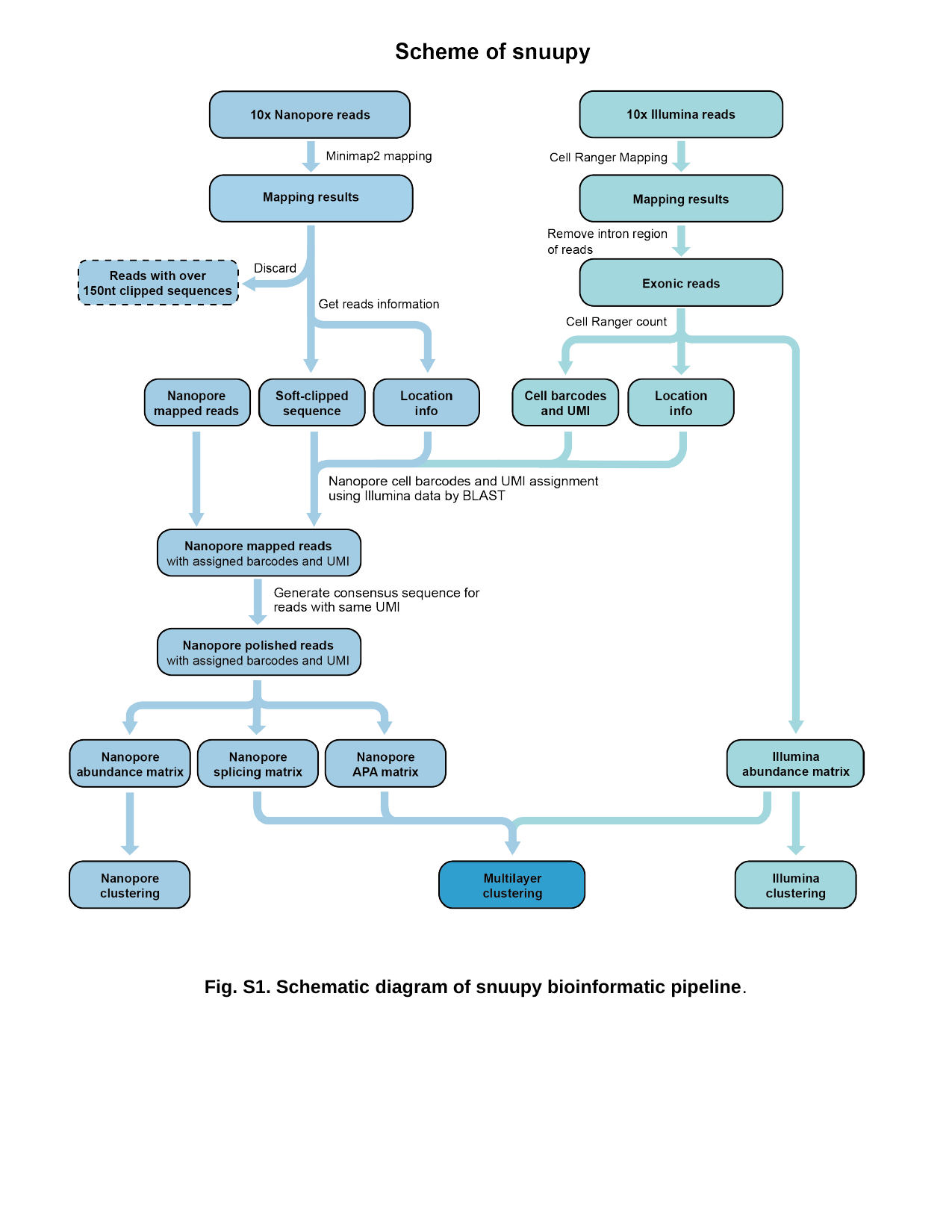

Fig. S1. Schematic diagram of snuupy bioinformatic pipeline.

## Slide 2
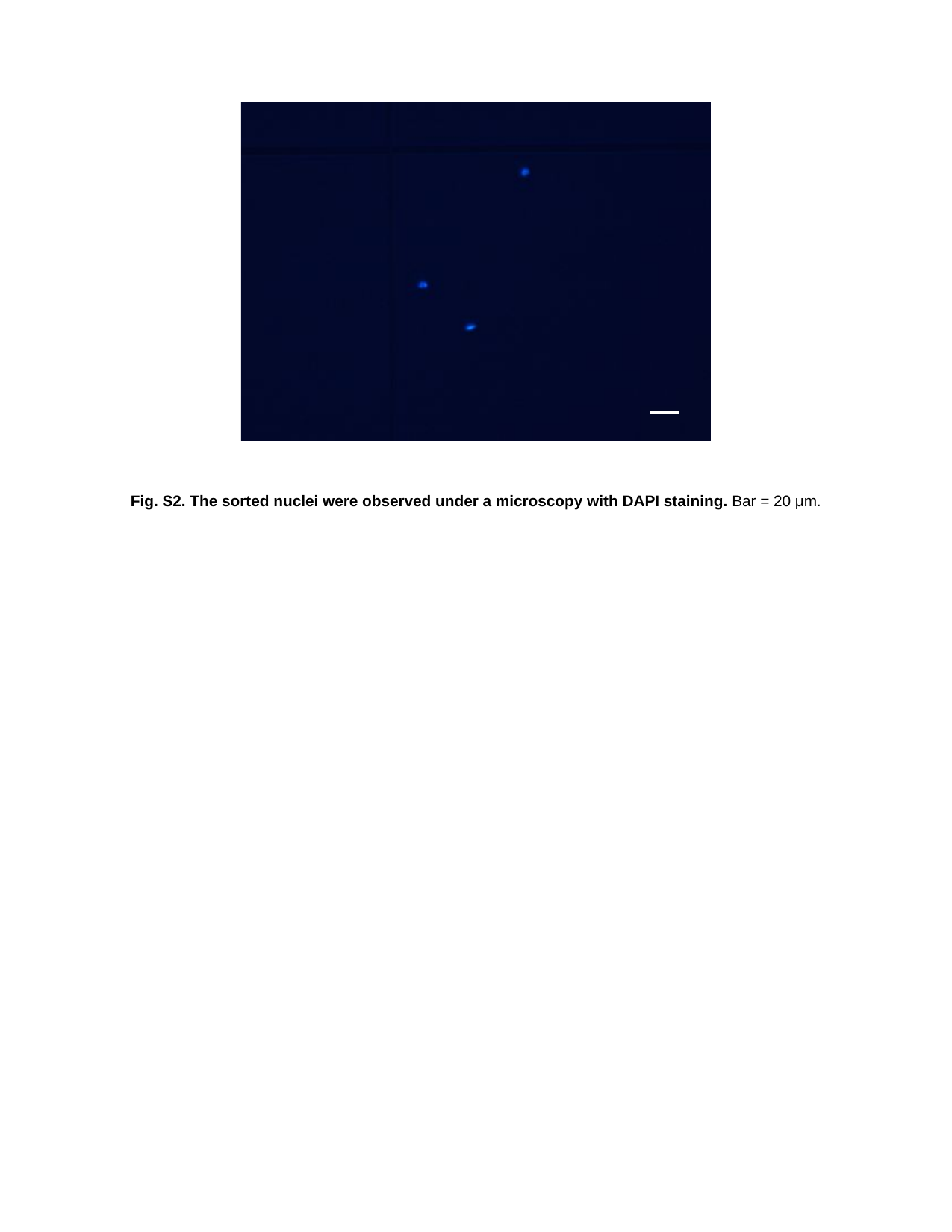

Fig. S2. The sorted nuclei were observed under a microscopy with DAPI staining. Bar = 20 μm.

## Slide 3
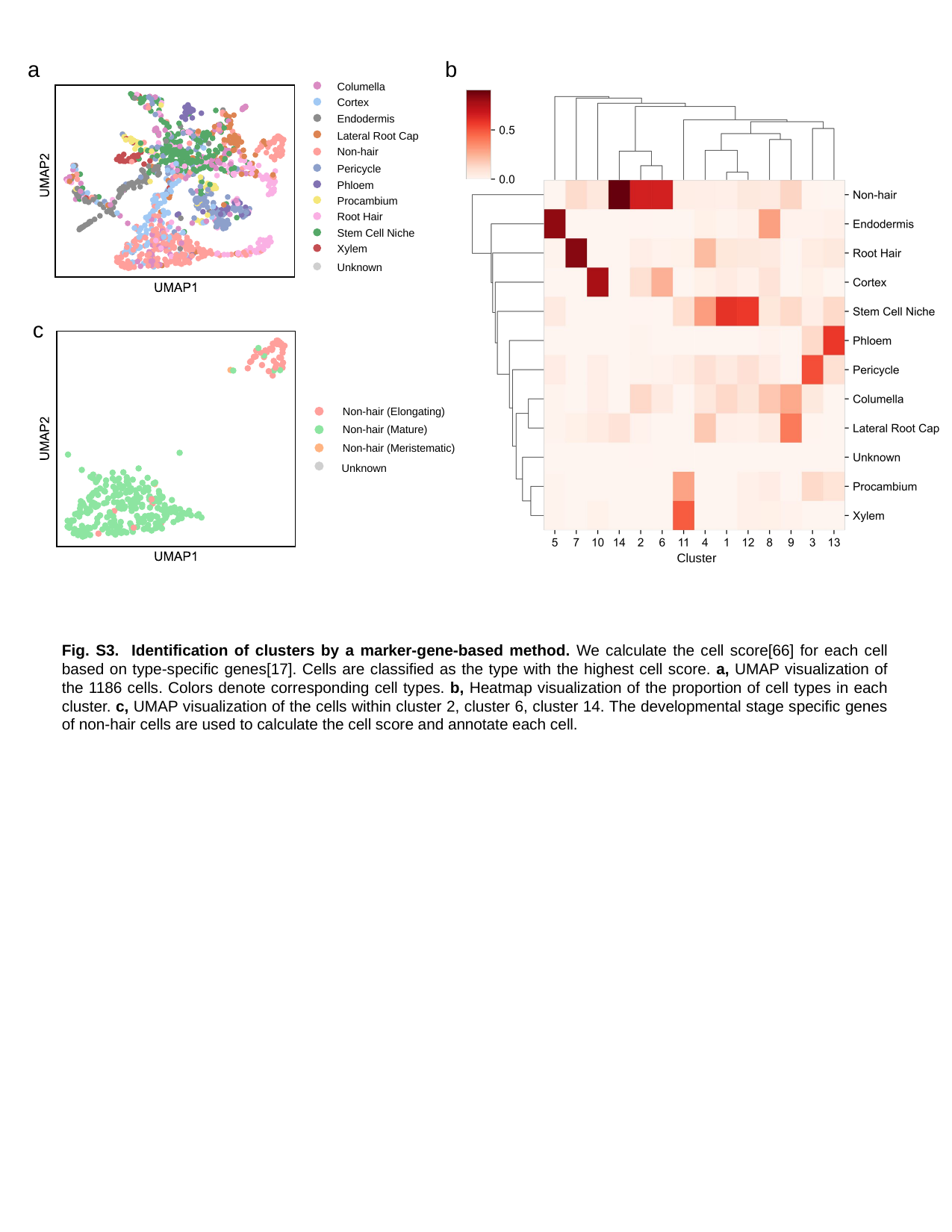

a
b
Columella
Cortex
Endodermis
Lateral Root Cap
Non-hair
Pericycle
Phloem
Procambium
Root Hair
Stem Cell Niche
Xylem
Unknown
c
Non-hair (Elongating)
Non-hair (Mature)
Non-hair (Meristematic)
Unknown
Cluster
Fig. S3. Identification of clusters by a marker-gene-based method. We calculate the cell score[66] for each cell based on type-specific genes[17]. Cells are classified as the type with the highest cell score. a, UMAP visualization of the 1186 cells. Colors denote corresponding cell types. b, Heatmap visualization of the proportion of cell types in each cluster. c, UMAP visualization of the cells within cluster 2, cluster 6, cluster 14. The developmental stage specific genes of non-hair cells are used to calculate the cell score and annotate each cell.

## Slide 4
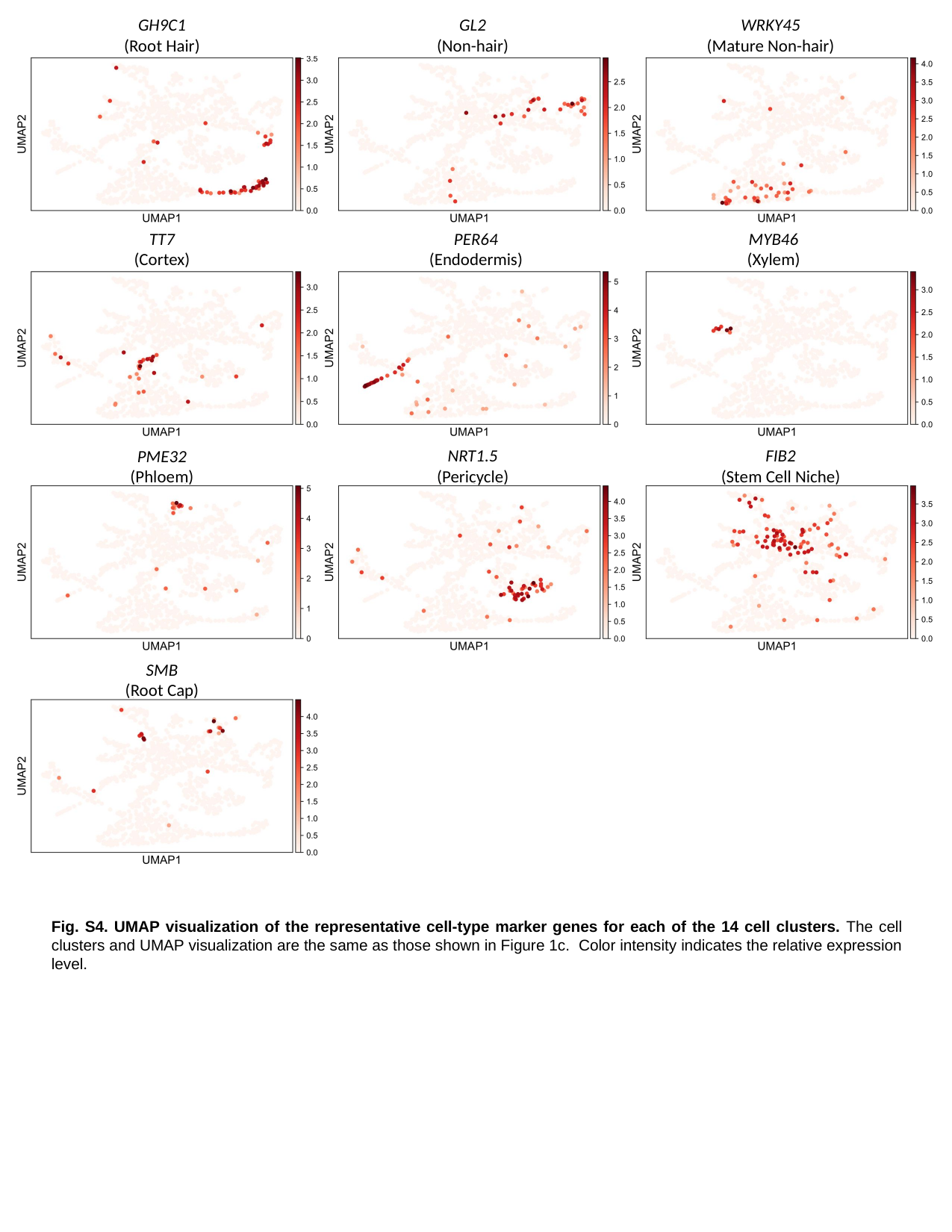

GH9C1
(Root Hair)
GL2
(Non-hair)
WRKY45
(Mature Non-hair)
TT7
(Cortex)
PER64
(Endodermis)
MYB46
(Xylem)
NRT1.5
(Pericycle)
FIB2
(Stem Cell Niche)
PME32
(Phloem)
SMB
(Root Cap)
Fig. S4. UMAP visualization of the representative cell-type marker genes for each of the 14 cell clusters. The cell clusters and UMAP visualization are the same as those shown in Figure 1c. Color intensity indicates the relative expression level.

## Slide 5
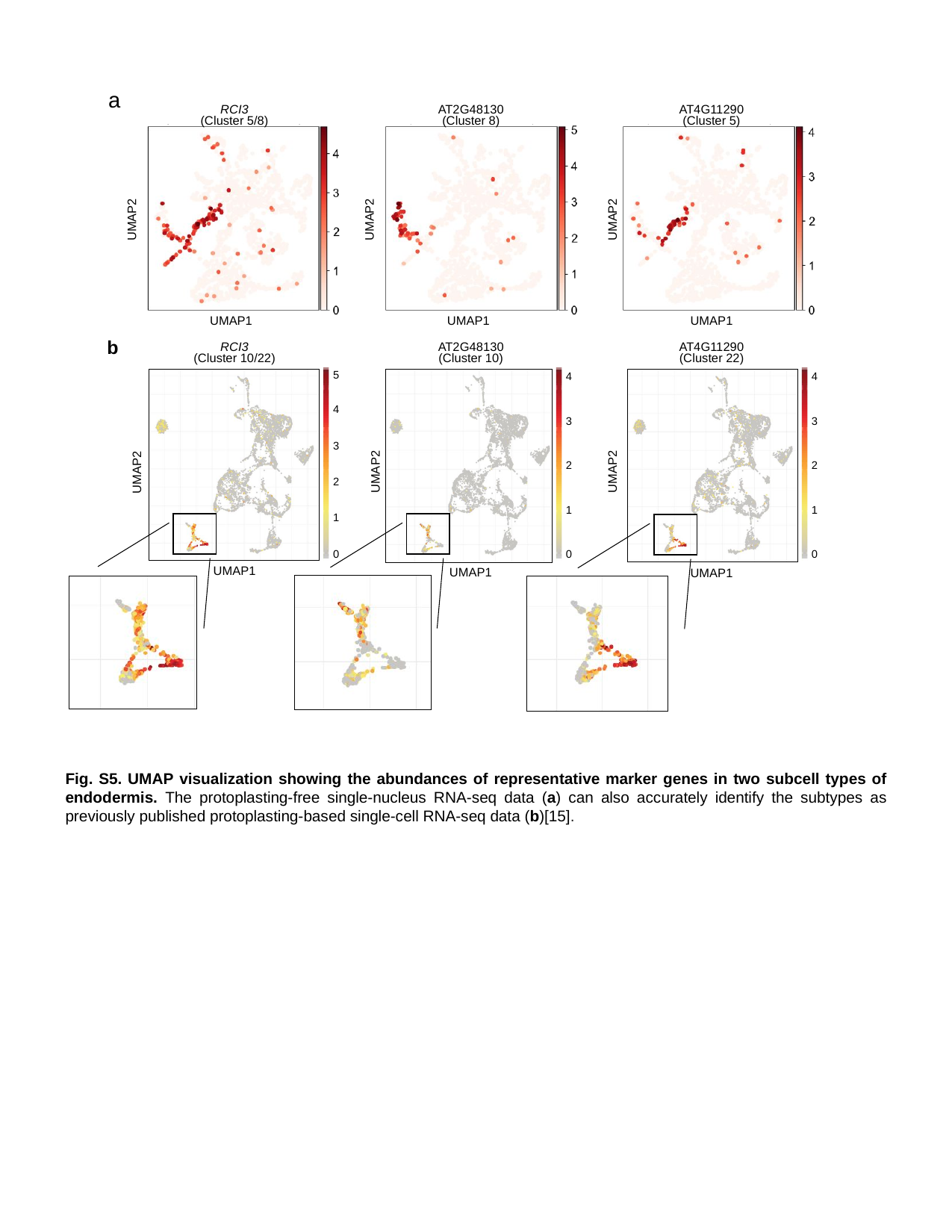

a
RCI3
(Cluster 5/8)
AT2G48130
(Cluster 8)
AT4G11290
(Cluster 5)
UMAP2
UMAP2
UMAP2
UMAP1
UMAP1
UMAP1
b
RCI3
(Cluster 10/22)
AT2G48130
(Cluster 10)
AT4G11290
(Cluster 22)
5
4
4
4
3
3
3
2
2
UMAP2
UMAP2
UMAP2
2
1
1
1
0
0
0
UMAP1
UMAP1
UMAP1
Fig. S5. UMAP visualization showing the abundances of representative marker genes in two subcell types of endodermis. The protoplasting-free single-nucleus RNA-seq data (a) can also accurately identify the subtypes as previously published protoplasting-based single-cell RNA-seq data (b)[15].

## Slide 6
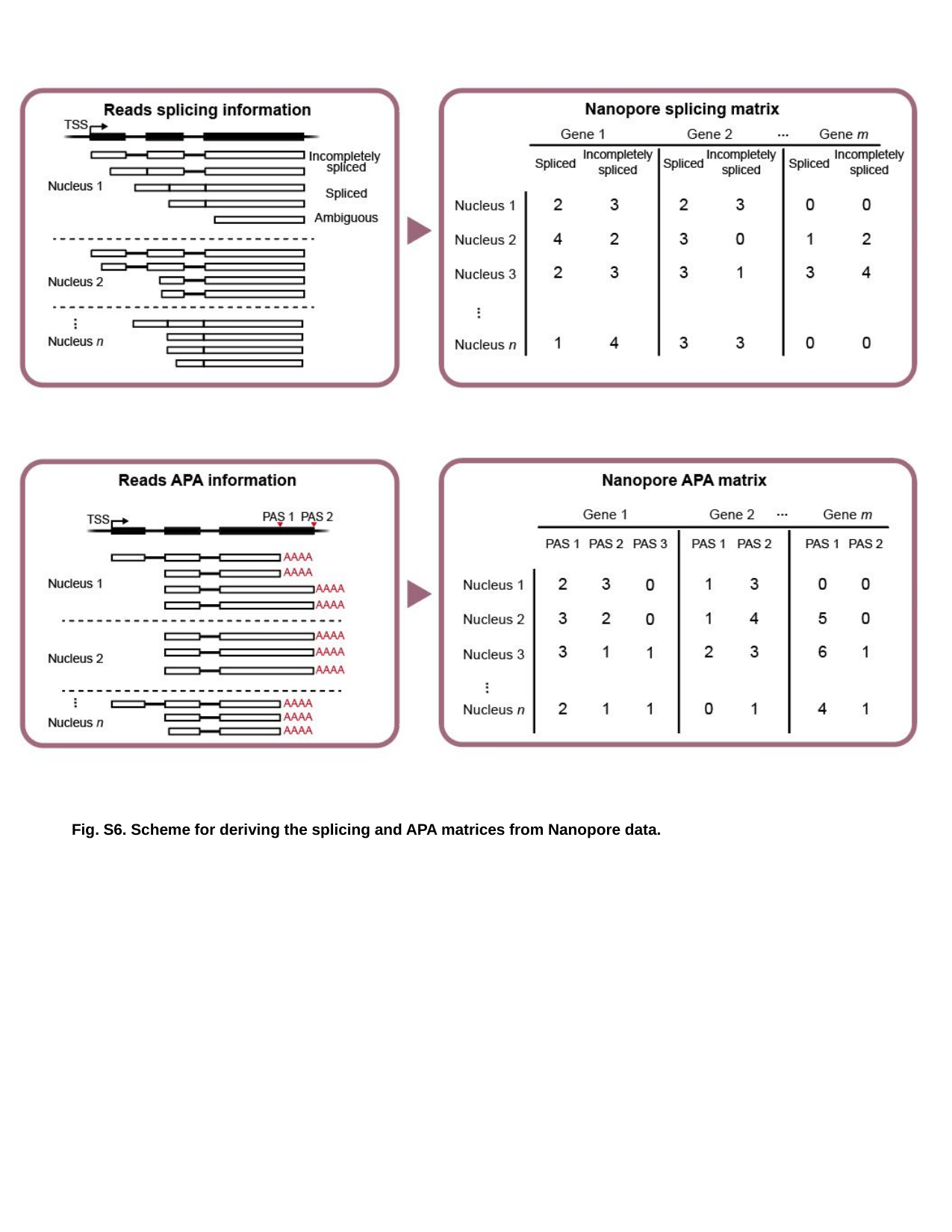

Fig. S6. Scheme for deriving the splicing and APA matrices from Nanopore data.

## Slide 7
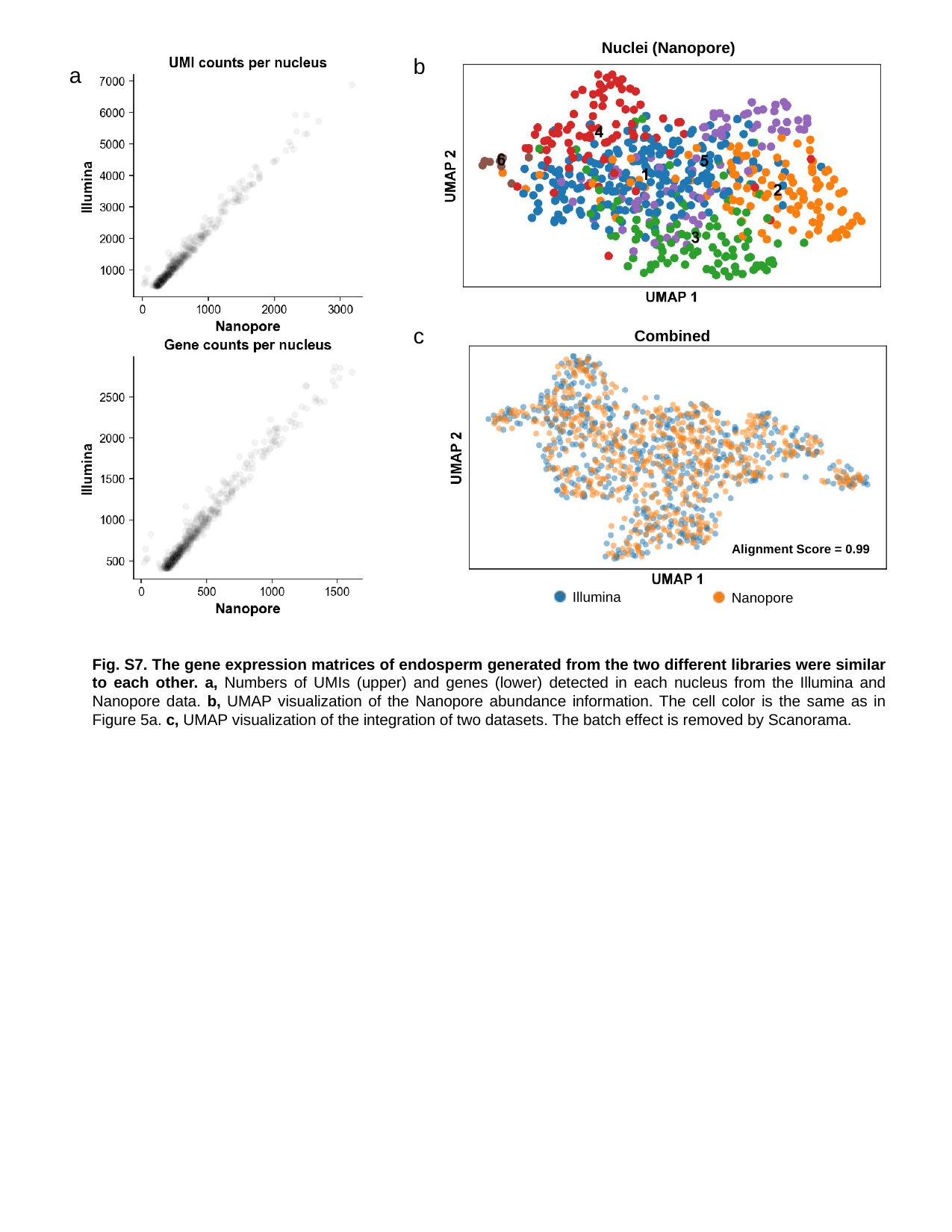

Nuclei (Nanopore)
b
a
c
Combined
Alignment Score = 0.99
Illumina
Nanopore
Fig. S7. The gene expression matrices of endosperm generated from the two different libraries were similar to each other. a, Numbers of UMIs (upper) and genes (lower) detected in each nucleus from the Illumina and Nanopore data. b, UMAP visualization of the Nanopore abundance information. The cell color is the same as in Figure 5a. c, UMAP visualization of the integration of two datasets. The batch effect is removed by Scanorama.
