# Supplementary material for: FlsnRNA-seq: protoplasting-free full-length single-nucleus RNA profiling in plants
Source: Genome Biol. 2021 Feb 19;22:66. doi: 10.1186/s13059-021-02288-0 (PMC7893963; doi:10.1186/s13059-021-02288-0)
Supplement: Supplementary file 3 — Additional file 3. Review history. [file 13059_2021_2288_MOESM3_ESM.docx]

Review History

**First round of review**

**Reviewer 1**

**Were you able to assess all statistics in the manuscript, including the appropriateness of statistical tests used?**

**No**

**Were you able to directly test the methods?**

**No**

**Comments to author:**

This is an important innovation. Single cell transcriptome analysis in plants has been hampered by the need to remove the cell wall and this has not been possible without the risk of significant changes in the transcriptome. The results presented suggest that this is a greatly improved strategy. Some estimation of the likely extent of changes in gene expression during the isolation of nuclei would be useful. The extent to which the method might be successful across a wide range of plant tissues might also be useful. Isolation of nuclei from some cell types may be much more difficult.

**Reviewer 2**

**Were you able to assess all statistics in the manuscript, including the appropriateness of statistical tests used?**

**Yes**

**Were you able to directly test the methods?**

**Yes**

**Comments to author:**

As we know, the main obstacle of single cell sequencing analysis used in plants is the practical difficulty of protoplast extraction, in which the removal of cell wall of plant cells is not easily controlled for different cell types. In this paper, Long and colleagues developed a nucleus-based, i.e. protoplast-free, system to evaluate the transcriptomes by combining both Illumina short reads and Nanopore long reads analysis from the same sample in plants. The authors first used Arabidopsis roots to validate the efficacy of their strategy. Compared to several publicly available single cell sequencing datasets using roots, and nuclear transcriptomes faithfully retain cell identity information. Moreover, by combining 10x Genomics and Nanopore long-read sequencing analysis, the authors show that information on splicing status and alternative polyadenylation could further improve the characterization of cell types. Subsequently, the authors demonstrate that this method is also applicable in the endosperm nuclei. Based on the feasibility and potential broad utility of this method in single cell sequencing technology, I recommend this manuscript for publication in this journal.

Two suggestions are listed as below.

1. The authors emphasize that a high proportion of incompletely spliced transcripts in the Cluster 4 of endosperm nuclei is indicative of active transcription. Considering that other possibilities may also explain this phenomena, such as delayed pre-mRNA decay or disrupted intron turnover rate, I suggest that the authors tune down this wording.

2. It may be more informative if the costs between the traditional protoplast-based method and this protoplast-free method can be provided.

3. The paper "Endosperm‑specific transcriptome analysis by applying the INTACT system" published in Plant Reproduction already showed that the nuclear transcriptome and total transcriptome of the endosperm are well correlated. The citation of this paper should further support the conclusion.

A typo:

1. "AAA" and "AAA" of PAS2 were overlayed in Fig. 4a, a statistical test should be done to support that the phenomena observed in this study is insensitive to major changes in growth conditions.

**Authors Response**

**Point-by-point responses to the reviewers’ comments:**

Reviewer comments:

Reviewer #1:

This is an important innovation. Single cell transcriptome analysis in plants has been hampered by the need to remove the cell wall and this has not been possible without the risk of significant changes in the transcriptome. The results presented suggest that this is a greatly improved strategy.

*Reply: We thank the reviewer for the positive comments.*

Some estimation of the likely extent of changes in gene expression during the isolation of nuclei would be useful.

*Reply: We thank the reviewer for this important suggestion. We have added a new reference (Del Toro-De León et al., Plant Reproduction, 2019) demonstrating that the transcriptome of isolated nuclei is well correlated with the total RNA transcriptome of the. We have revised the discussion to reflect this update.*

The extent to which the method might be successful across a wide range of plant tissues might also be useful. Isolation of nuclei from some cell types may be much more difficult.

*Reply: We fully agree with the reviewer on this important comment. While nuclei isolation is more widely applicable compared to protoplasting, it still remains challenging to obtain nuclei from certain cell types and thus requires further refinements of the nuclei isolation protocol. We have added a recent reference on protocol for isolating nuclei for single-cell sequencing in plant (Thibivilliers et al., Current Protocol in Plant Biology, 2020). We have revised the discussion to reflect this update.*

Reviewer #2:

As we know, the main obstacle of single cell sequencing analysis used in plants is the practical difficulty of protoplast extraction, in which the removal of cell wall of plant cells is not easily controlled for different cell types. In this paper, Long and colleagues developed a nucleus-based, i.e. protoplast-free, system to evaluate the transcriptomes by combining both Illumina short reads and Nanopore long reads analysis from the same sample in plants. The authors first used Arabidopsis roots to validate the efficacy of their strategy. Compared to several publicly available single cell sequencing datasets using roots, and nuclear transcriptomes faithfully retain cell identity information. Moreover, by combining 10x Genomics and Nanopore long-read sequencing analysis, the authors show that information on splicing status and alternative polyadenylation could further improve the characterization of cell types. Subsequently, the authors demonstrate that this method is also applicable in the endosperm nuclei. Based on the feasibility and potential broad utility of this method in single cell sequencing technology, I recommend this manuscript for publication in this journal.

*Reply: We thank the reviewer for the positive comments.*

Two suggestions are listed as below. 1. The authors emphasize that a high proportion of incompletely spliced transcripts in the Cluster 4 of endosperm nuclei is indicative of active transcription. Considering that other possibilities may also explain this phenomena, such as delayed pre-mRNA decay or disrupted intron turnover rate, I suggest that the authors tune down this wording.

*Reply: We thank the reviewer for raising this important point and agree that other possibilities could also explain the observed high proportion of incompletely spliced transcripts. We have therefore revised the manuscript and tuned down the statements at two places when this phenomenon was mentioned. Please see below for the updated text: “The high ratio of incompletely spliced transcripts in this particular cluster of endosperm nuclei may reflect delayed pre-mRNA decay, disrupted intron turnover rate, or a global activation of transcription.” “our method identified a unique cluster of endosperm nuclei with a high proportion of incompletely spliced transcripts, and further investigation could determine whether this is due to increase in transcription or delay in splicing.”*

2. It may be more informative if the costs between the traditional protoplast-based method and this protoplast-free method can be provided.

*Reply: We thank the reviewer for this suggestion and this is an important factor of consideration when deciding which approach to use. Procedure wise, the nuclei approach saves the cost on protoplasting but requires FACS, so the cost is comparable between nuclei isolation and protoplasting. In term of cost per cell, the standard 10x Genomics protocol is relatively more efficient in capturing the protoplasted cell than the much smaller nucleus, resulted in a higher cost per cell for the nuclei approach, a step which would benefit from further optimization and adjustment of the 10x Genomics protocol. We have added a sentence to discuss the cost for single cell sequencing in plant using isolated nuclei verses using protoplasts: “The cost per cell for single-cell sequencing using isolated nuclei on the 10x Genomics platform is still relatively higher compared to using protoplast due to a lower capturing efficiency of 10x Genomics on nuclei verses cell, possibly due to the much smaller size of nucleus, thus this step still could benefit from further optimization.”*

3. The paper "Endosperm specific transcriptome analysis by applying the INTACT system" published in Plant Reproduction already showed that the nuclear transcriptome and total transcriptome of the endosperm are well correlated. The citation of this paper should further support the conclusion.

*Reply: We thank the reviewer for suggesting this highly relevant reference. We have cited this paper in the revised manuscript to support the utility of nuclear transcriptome: “In plants, the transcriptome from isolated nuclei and from total RNA of the endosperm have also been shown to be well correlated [41].”*

A typo: 1. "AAA" and "AAA" of PAS2 were overlayed in Fig. 4a, a statistical test should be done to support that the phenomena observed in this study is insensitive to major changes in growth conditions.

*Reply: We thank the reviewer for catching this and our apology for the error. The overlayed text in Fig. 4a has been fixed. We have also added statistical tests for the results shown in Fig. 4c and Fig. 5c and updated the figure legends accordingly.*
